# Supplementary material for: The causal role of circulating inflammatory markers in osteoporosis: a bidirectional Mendelian randomized study
Source: Front Immunol. 2024 Jul 18;15:1412298. doi: 10.3389/fimmu.2024.1412298 (PMC11291241; doi:10.3389/fimmu.2024.1412298)
Supplement: Supplementary file 1 [file DataSheet_1.zip › supplementary material/OSTEOPOROSIS-CIM/GCST90274762.tsv.gz_leave_one_out.pdf]

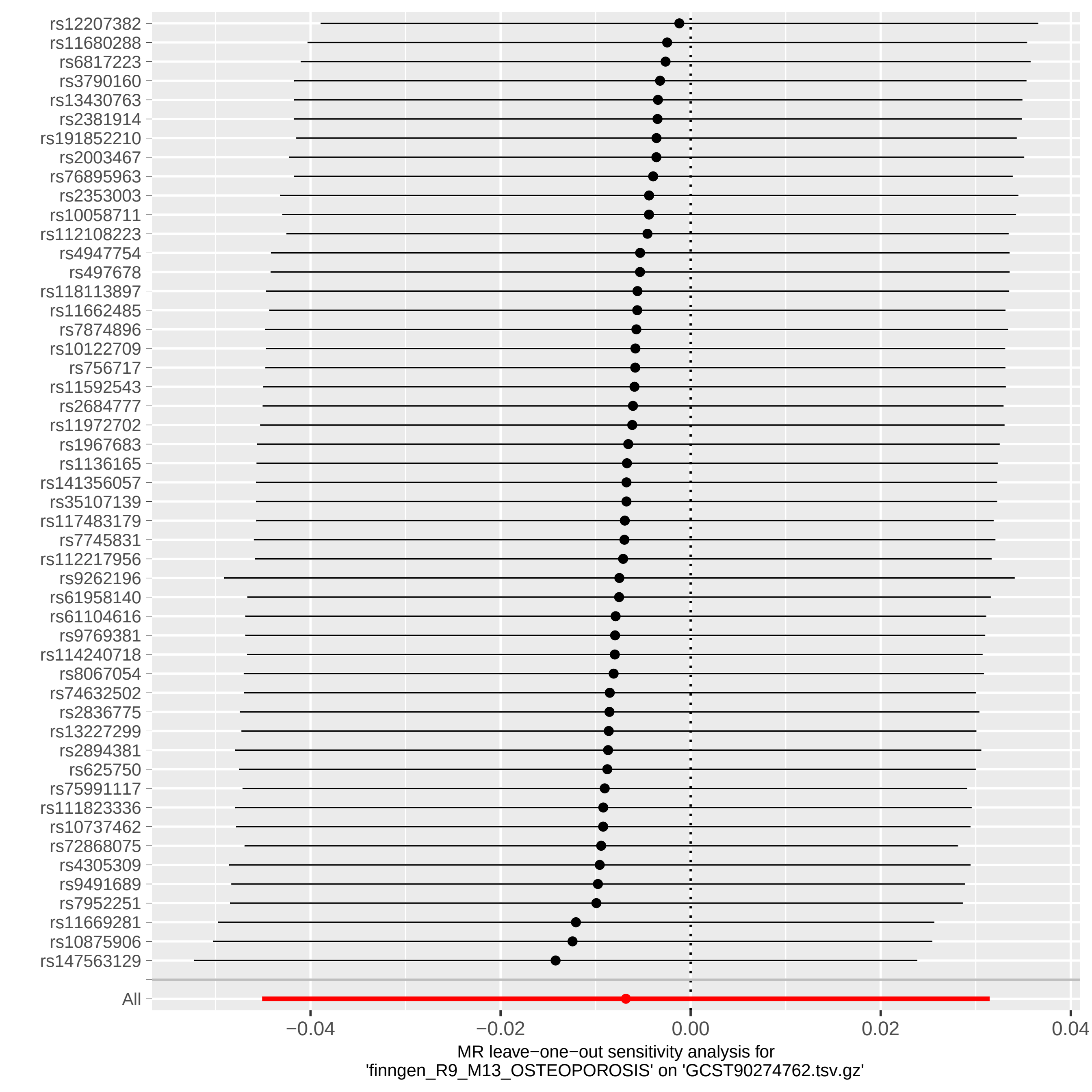

rs12207382
rs11680288
rs6817223
rs3790160
rs13430763
rs2381914
rs191852210
rs2003467
rs76895963
rs2353003
rs10058711
rs112108223
rs4947754
rs497678
rs118113897
rs11662485
rs7874896
rs10122709
rs756717
rs11592543
rs2684777
rs11972702
rs1967683
rs1136165
rs141356057
rs35107139
rs117483179
rs7745831
rs112217956
rs9262196
rs61958140
rs61104616
rs9769381
rs114240718
rs8067054
rs74632502
rs2836775
rs13227299
rs2894381
rs625750
rs75991117
rs111823336
rs10737462
rs72868075
rs4305309
rs9491689
rs7952251
rs11669281
rs10875906
rs147563129
All
−0.04
−0.02
0.00
0.02
0.04
MR leave−one−out sensitivity analysis for
'finngen_R9_M13_OSTEOPOROSIS' on 'GCST90274762.tsv.gz'
